# Study on the development characteristics and spatial and temporal patterns of population ageing in 31 central cities in China

**DOI:** 10.3389/fpubh.2024.1341455

**Published:** 2024-04-18

**Authors:** Le Zhang, Hengna Ren, Chen Li

**Affiliations:** ^1^School of Marxism, Jiangnan University, Wuxi, China; ^2^Business School, University of Shanghai for Science and Technology, Shanghai, China; ^3^School of Marxism, Shanghai University of Engineering Science, Shanghai, China; ^4^School of Management, Shanghai University of Engineering Science, Shanghai, China

**Keywords:** population ageing, dynamic characteristics, central cities, regional differences, quadrant analysis

## Abstract

**Background:**

Population ageing is inseparable from technological innovation, social progress and the development of human civilization, and constitutes a new element in the development of contemporary human history.

**Objective:**

To dynamically analyses the developmental, structural and growth characteristics of population ageing in 31 provincial capitals and municipalities in China, using the data of the fifth national census in 2000 and the seventh national census in 2020.

**Methods:**

The development characteristics and spatial and temporal patterns of population aging in the 31 cities were measured using the population aging index growth model, Theil’s index, coefficient of variation, population aging index and other analytical methods.

**Results:**

(1) From 2000 to 2020, the population aging rate of the 31 central cities generally increased, and the population aging level of the cities showed the characteristics of “East-Central-Northeast-West” to “Northeast-East-Central-West” decreasing. (2) Regional differences in the ratio of old to young are relatively high, while regional differences in the level of population ageing are relatively small. The level of population ageing is classified with the indicators of size structure, family structure and age structure in the first and third quadrants, and with the geographic concentration rate in the second and fourth quadrants. (3) China’s population ageing has a T-shaped spatial distribution characteristic pointing along the coast - along the Yangtze Rivers.

**Conclusion:**

The 31 central cities are the center of gravity of China’s economy and have strong economic power in dealing with the challenges of population ageing, but how to make population ageing compatible with the economy and society, and then promote sustainable population development, is a topic that needs further attention in the study.

## 1 Introduction

In today’s world, economically developed countries have largely entered the ranks of old-age countries, and the trend of population ageing in developing countries is increasing (1). As an inevitable trend of the global demographic transition, population ageing is governed by the laws of human social development, and the significant reduction of mortality rates in childhood and at birth, as well as mortality rates from infectious diseases, has pushed forward the extension of life expectancy in low- and middle-income countries, and the increasing life expectancy has contributed to the increasing proportion of the older adult population (2, 3), that is to say, the level of population ageing is constantly rising. The salient social feature of population ageing is the upward shift in the centre of gravity of the age structure, with the size of the older adult population gradually expanding, the size of the working-age population tending to shrink, and the size of the child population continuing to decline (4). The direct cause of population ageing is the change of birth rate and death rate, and the population structure experiences the process of high birth rate, high death rate and low natural growth rate to high birth rate, low death rate and high natural growth rate, and then to low birth rate, low death rate and low natural growth rate (5), which is the result of the quantitative change of population structure to the qualitative leap.

**Figure 1:**
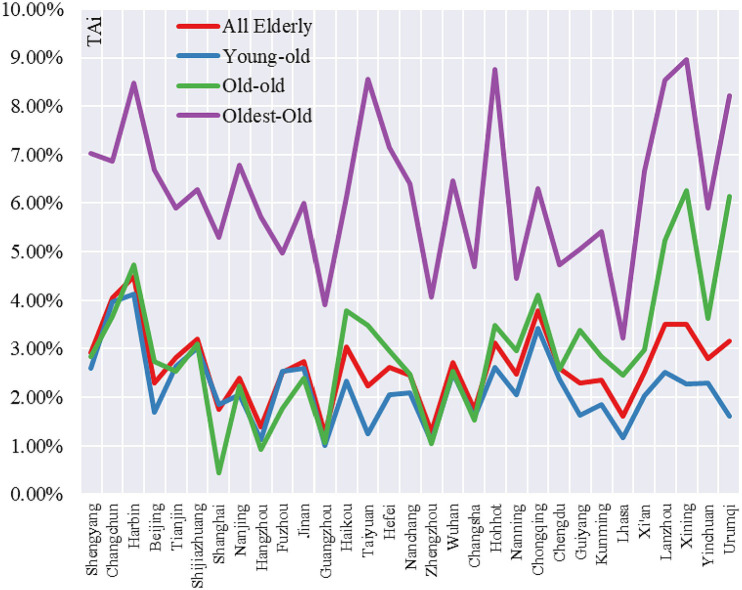
Tempo of Elderly Age Concentration by province in China.

According to the data of China’s seventh population census in 2020, the total fertility rate of Chinese women of childbearing age has been reduced to 1.30 (6), and in 2022, it will further drop to 1.08, far below the warning line of 1.5 that is commonly used by the international community, and the proportion of the population aged 65 years and above has reached 13.50%, which is close to the warning line of 14% of the international community that is commonly used by the international community for the moderate aging, and it is estimated that in 2050, the number of people aged 65 years and above will reach 477 million, which will account for 34.9% of the total population. Population aged 65 and above will reach 477 million by 2050, accounting for 34.9% of the total population, implying that China will face an even more severe challenge of population aging in the future (7). Due to the large size of China’s population and the differences in the level of economic development, social system, and historical trajectory of China’s development from that of developed countries, China’s low fertility rate and population aging in this unique context make it impossible for any coping strategies to be learnt from the past (8). 31 provincial capitals and municipalities represent China’s level of production and development in a certain way, and the population, as an important component of the sustainable development of the cities, is a key factor in analysing the change of their age structure, especially the ageing of the population. As the population is an important component of the sustainable development of cities, analysing its age structure, especially the trend of population aging, is of great value in grasping the law of economic and social development of the urban population and promoting the sustainable development of the population (Figure 1).

Discussions on the spatial and temporal patterns and developmental characteristics of population ageing have been conducted in three main areas. First, the study of the current situation of the spatio-temporal pattern and development characteristics of population ageing. Using population gravity model, spatial autocorrelation, semi-variance function and other regional difference analysis methods (9–13), the spatial–temporal pattern of population aging or aging in China, local provinces and municipalities is revealed through indicators such as the coefficient of the young, the coefficient of the old, the old-to-young ratio, the coefficient of the young dependency, the coefficient of the old dependency, and so on (14–17). Second, the explanation of the factors affecting the spatio-temporal pattern of population aging. On the one hand, correlation analysis, geographic detector and other analytical methods are used to reveal the influencing factors of population aging (18–21); on the other hand, the phenomenon of population aging is explained mechanistically. For the aging phenomenon, it is mainly the result of the increasing life expectancy of the population due to the combination of material living standards, quality of living environment, and improvement of medical and health technology (22, 23). Thirdly, research on countermeasures for the temporal and spatial patterns of population ageing. Many countries in the world are facing the challenge of population aging (24–29), and governments are trying to do everything they can to provide security for low fertility and old-age services (30–32), and development concepts such as productive aging, healthy aging, and active aging are becoming increasingly popular (33–35), which provide reference ideas for the solution of regional population problems.

**Figure 2:**
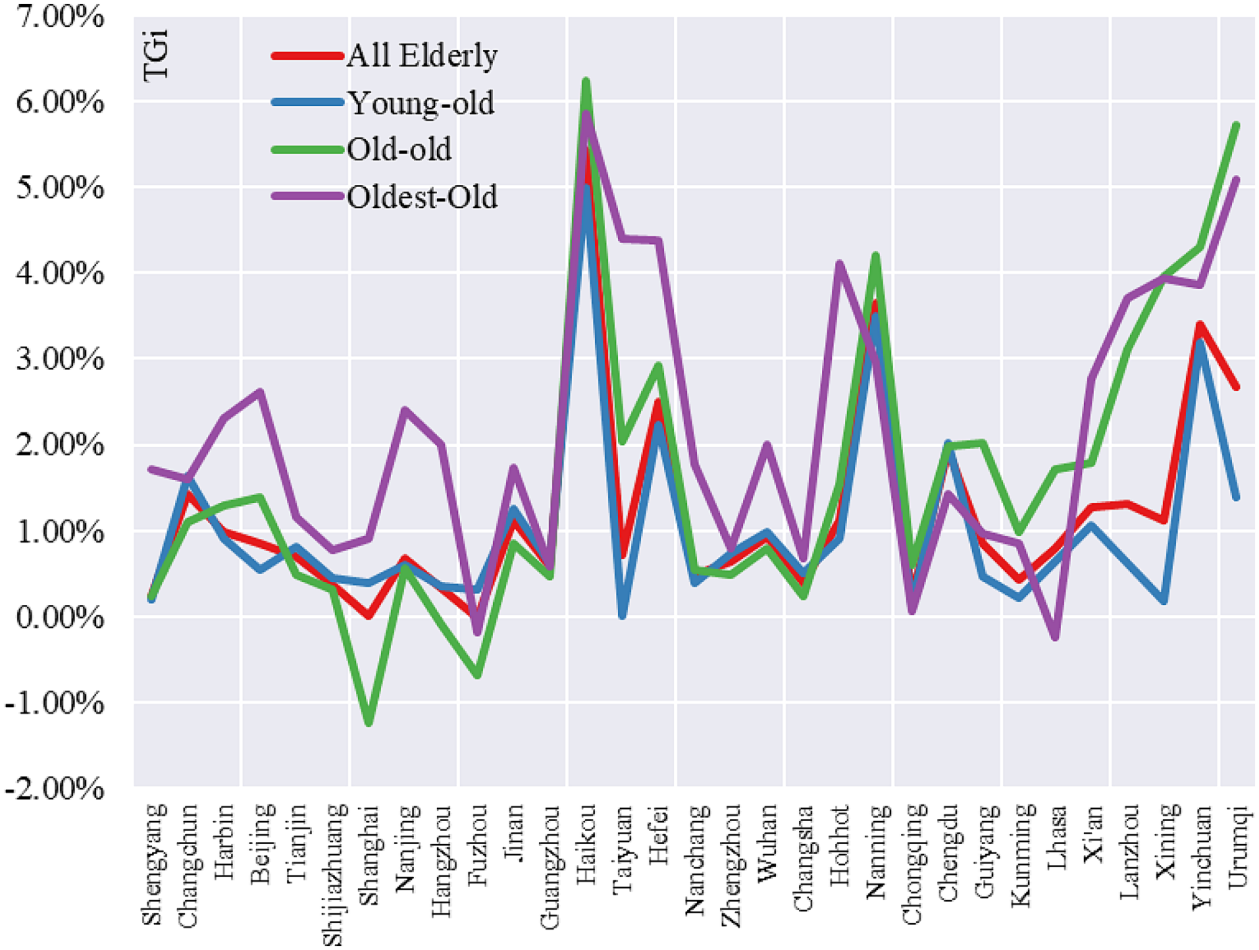
Tempo of Elderly Geographical Concentration by province in China.

In summary, academic research on the development characteristics and spatial and temporal patterns of population ageing is quite rich, but there are also areas that are worthy of further debate. Firstly, with regard to the indicators and comprehensive measurement of population ageing, it focuses on the analysis of a single indicator of population ageing and neglects the comprehensive analysis of the structure, scale and speed of population ageing. As population ageing is a complex economic and social phenomenon, considering only the measurement of a single indicator of population ageing inevitably leads to generalization and lack of multi-dimensional measurement of population ageing, we will conduct a comprehensive analysis of multiple indicators of population ageing in order to examine the issue of population ageing in a more comprehensive manner. Secondly, many studies have revealed the spatial and temporal patterns of population ageing at the provincial, prefectural, and county levels, but not enough has been done on population ageing in China’s 31 provincial capitals and municipalities, which to some extent represent China’s level of productive development, and analyses of changes in their age structures, especially trends in population ageing, are useful for exploring the issue of population ageing in China. The analysis of their age structure change, especially the trend of population aging, is typical and representative for the discussion of population aging in China, and we will reveal its multiple dynamic characteristics from the perspectives of population aging level, structure, and growth, so as to provide an empirical basis for the revelation of the general law of population aging (Figure 2).

## 2 Research design

### 2.1 Research methods

#### 2.1.1 Population ageing rate and classification criteria

The population ageing rate—that is, the level of population ageing (or the coefficient of old age) —is a measure of the proportion of older population to the total population. In accordance with international practice and academic consensus, it is measured as the percentage of the population aged 65 and older in the total population using the following formula:

(1) $$par=\frac{p_{65+}}{p}\times 100\%$$

In Equation (1), *par* denotes the population ageing rate, *p65+* is the size of the regional population aged 65 and above, and *p* denotes the size of the overall regional population.

Normally, a population ageing rate of 7.0% is regarded as the basic criterion for entering an ageing society. However, there are differences in the refinement of the population ageing measurement criteria. The research group ‘Research on the Development Indicator System of the Aging Career’ classified the population ageing rate of 7.0–14.0%, 14.0–22.0 and 22.0% and above into three types of low-aging, medium-aging and severe-aging in that order, although there are no subdivisions of other age groups (33). Wang classified the age structure of the population into six types: population ageing rate lower than 4.0% is called the young type, 4.0–5.5% is adult Type I, 5.5–7.0% is adult Type II, 7.0–10.0% is old-age Type I, 10.0–14.0% is old-age Type II, and more than 14.0% is old age Type III. There is no classification for 14.0% and above (34). In this paper, we combine the above two divisions to classify the population ageing rate into less than 4.0% as young, 4.0–7.0% as adult, 7.0–10.0% as low-old-age Type I, 10.0–14.0% as low-old-age Type II, 14.0–18.0% as medium-old-age Type I, and 18.0–22.0% as medium-old-age Type II and 22.0% and above severe geriatric type.

#### 2.1.2 Exponential growth modeling of population ageing

The rate of population ageing varies in different regions, and an exponential growth model can dynamically measure the evolution of population ageing. This article draws on the exponential growth model of population ageing used in Wu’s empirical analysis to measure the age concentration rate and the geographic concentration rate of the older people using the following formulae (35):

(2) $$P\left(t+n\right)\left(65+\right)=e^{n\times r\left(65+\right)}\times P\left(t\right)\left(65+\right)$$

(3) $$r\left(65+\right)=\frac{1}{n}\times \ln\left[\frac{P\left(t+n\right)\left(65+\right)}{P\left(t\right)\left(65+\right)}\right]$$

(4) $$I\left(t+n\right)\left(65+\right)=e^{n\times TAi\left(65+\right)}\times I\left(t\right)\left(65+\right)$$

(5) $$SAi\left(t+n\right)\left(65+\right)=e^{n\times TGi\left(65+\right)}\times SAi\left(t\right)\left(65+\right)$$

(6) $$\begin{array}{l} TAi\left(65+\right)=\frac{1}{n}\times \ln\left[\frac{P_{i}\left(t+n\right)\left(65+\right)}{P_{i}\left(t\right)\left(65+\right)}\right]-\frac{1}{n}\times \ln\left[\frac{P_{i}\left(t+n\right)\left(0+\right)}{P_{i}\left(t\right)\left(0+\right)}\right]\\ \;=r_{i}\left(65+\right)-r_{i}\left(0+\right) \end{array}$$

(7) $$\begin{array}{l} TGi\left(65+\right)=\frac{1}{n}\times \ln\left[\frac{P_{i}\left(t+n\right)\left(65+\right)}{P_{i}\left(t\right)\left(65+\right)}\right]-\frac{1}{n}\times \ln\left[\frac{P\left(t+n\right)\left(65+\right)}{P\left(t\right)\left(65+\right)}\right]\\ \;=r_{i}\left(65+\right)-r\left(65+\right) \end{array}$$

In Equations (2–7), *P(t)(65+)* is the proportion of urban older population (65 years old and above) in year *t* (base period) and P*(t + n)(65+)* is the number of older people in China after *n* years; *r(65+)* is the average annual growth rate of the older people population in China; *ri(65+)* is the average annual growth rate of the older population in region *i*; *ri(0+)* is the average annual growth rate of the total population in region *i*; *I(t)(65+)* is the share of region *i*’s older people population in the total population of the region in the base period; *I(t + n)(65+)* is the share of region *i*’s older people population in the total population of the region after *n* years; *SAi(t)(65+)* is the share of region *i*’s older population in the national older population in the base period; *SAi(t + n)(65+)* is the share of region *i*’s older people population in the national older population after *n* years; *Pi (t)(65+)* is the number of older persons in region *i* in the base period; *Pi(t + n)(65+)* is the number of older persons in region *i* after n years; *Pi(t)(0+)* is the total population of region *i* in the base period; *Pi(t + n)(0+)* is the total population of region *i* after *n* years; *TAi(65+)* is the old-age concentration rate and *TAi(65+)* > 0 (<0 or = 0), indicating that ageing in city *i* is ahead (lagging or flat) at the national level.

#### 2.1.3 Measuring regional differences in population ageing

##### 2.1.3.1 Theil index

Drawing on the analytical ideas of regional comparisons and spatial–temporal variation (36–39), the article will use the Theil index to examine inequality and difference in terms of the concepts of informativeness and entropy. The Theil index examines inequality and variability using the concepts of informativeness and entropy, decomposes the overall variability into variability between parts and within parts, and has a wide range of applications in analysing and decomposing variability. The composite entropy index examines the variability between individuals based on the concepts of informativeness and entropy and is the expected value of informativeness, that is, the volume of expected information. The closer the relationship between individuals, the smaller the combined entropy index (40).

(8) $$GE=\{\begin{array}{l} \overset{n}{\underset{i=1}{\sum }}p_{i}\left[{\left(y_{i}/u\right)}^{c}-1\right],c\neq 0,1\\ \overset{n}{\underset{i=1}{\sum }}p_{i}\left(y_{i}/u\right)\mathrm{lg}\left(y_{i}/u\right),c=1\\ \overset{n}{\underset{i=1}{\sum }}p_{i}\mathrm{lg}\left(y_{i}/u\right),c=0 \end{array}$$

In the above equation, parameter *c* is used to determine the sensitivity of the index change. In general, this determines the sensitivity to an exponential change when *c* < 2. When *c* = 0 and 1, it is known as Theil’s index.

Owing to its ability to divide the overall variation into within-zone and between-zone variations, Theil’s index is widely used in empirical studies of overall spatial heterogeneity as well as spatial heterogeneity, and is calculated by the formula:

(9) $$\mathrm{Theil}=\overset{n}{\underset{i=1}{\sum }}T_{i}\ln\left({nT}_{i}\right)=T_{WR}+T_{BR}$$

Theil index can be further decomposed into intrazonal and interzonal differences if the study area is categorized into groups according to a specific methodology.

(10) $$\begin{array}{l} T_{WR}=\overset{n_{db}}{\underset{i=1}{\sum }}T_{i}\ln\left(n_{db}\frac{T_{i}}{T_{db}}\right)+\overset{n_{d}}{\underset{i=1}{\sum }}T_{i}\ln\left(n_{d}\frac{T_{i}}{T_{d}}\right)\\ \;+\overset{n_{z}}{\underset{i=1}{\sum }}T_{i}\ln\left(n_{z}\frac{T_{i}}{T_{z}}\right)+\overset{n_{x}}{\underset{i=1}{\sum }}T_{i}\ln\left(n_{x}\frac{T_{i}}{T_{x}}\right) \end{array}$$

(11) $$\begin{array}{l} T_{BR}=T_{db}\ln\left(T_{db}\frac{n}{n_{db}}\right)+T_{d}\ln\left(T_{d}\frac{n}{n_{d}}\right)\\ \;+T_{z}\ln\left(T_{z}\frac{n}{n_{z}}\right)+T_{x}\ln\left(T_{x}\frac{n}{n_{x}}\right) \end{array}$$

In the above formulae, Theil denotes Theil’s index; *n* is the number of provincial units within the sample region; *TWR* is the difference in provincial capital cities or municipalities within the four regional zones of the Northeast, East, Central and West; *TBR* is the difference in provincial capital cities or municipalities between the four regional zones. Furthermore, *ndb*, *nd*, *nz*, and *nx* are the number of provincial capital cities or municipalities in provincial units within the Northeast, East, Central and West regions, respectively; *ndb*, *nd*, *nz*, and *nx* represent the number of provincial capitals or municipalities in the provincial units in the Northeast, East, Central and West regions, respectively; *Ti* is the ratio of the measurements of provincial capitals or municipalities within the region to the average of the 31 central cities nationwide; and *Tdb*, *Td*, *Tz*, and *Tx* are the ratios of the measurements of provincial capitals or municipalities in the Northeast, East, Central and West regions, respectively, to the average of the 31 central cities nationwide. The three central cities in the Northeast region are Shenyang, Changchun and Harbin; the 10 central cities in the East region are Beijing, Tianjin, Shijiazhuang, Shanghai, Nanjing, Hangzhou, Fuzhou, Jinan, Guangzhou and Haikou; the six central cities in the Central region are Taiyuan, Hefei, Nanchang, Zhengzhou, Wuhan and Changsha; and the 12 central cities in the West region are Hohhot, Nanning, Chongqing, Chengdu, Guiyang, Kunming, Lhasa, Xi’an, Lanzhou, Xining, Yinchuan and Urumqi. The 12 centres in the western region are Hohhot, Nanning, Chongqing, Chengdu, Guiyang, Kunming, Lhasa, Xi’an, Lanzhou, Xining, Yinchuan and Urumqi.

##### 2.1.3.2 Coefficient of variation

The coefficient of variation (*CV*) is a statistical measure of the degree of variation of each observation in the information, and is an important indicator for measuring regional differences, which is measured by the following formula (41):

(12) $$CV=\frac{1}{\bar{h}}\sqrt{\overset{n}{\underset{i=1}{\sum }}\frac{\left(h_{i}-\bar{h}\right)}{\left(n-1\right)}}$$

In the above equation, *CV* is the coefficient of variation; *n* is the number of sample cities; *hi* is the population ageing indicator of city *i*; and is the average value of *hi*. The larger the coefficient of variation *CV*, the greater the difference.

#### 2.1.4 Composite measure of population ageing index

##### 2.1.4.1 Analytic hierarchy process

Saaty proposed the Analytic Hierarchy Process (AHP) in 1970, which is a systematic and hierarchical analytical method that combines qualitative and quantitative analyses. AHP divides the evaluation objectives into objective level (A), criterion level (B) and programme level (C). By comparing them two-by-two, the weight of each criterion on the objectives is determined, which is characterized by subjective assignment.

AHP usually uses a 1–9 scale to judge the relative importance of each indicator in the system being evaluated, thus creating a judgment matrix. Let the evaluation element be *X* = {x1,x2,…,xi,…,xn}, *xij* denotes the result of the comparison of the importance of *xi* relative to *xj*, and the 1–9 scale is used. Based on the meaning of the *xij* value scale, the Delphi method, which takes the average scoring of experts, is used to compare the elements of the assessment element set *X* two-by-two to obtain the judgment matrix *P*. The judgment matrix satisfies (42).

(13) $$P=X_{n\times n}$$

*xij* = *1/xji* in the judgment matrix *Xn×n*. The eigenvector *Mn×1*, corresponding to the maximum eigenvalue λ*max*, was measured using the equation *PM = λmax*, and the weight *wα* of each evaluation index was obtained using the normalized eigenvector *M*. This was measured using the sum-product method.

To determine the validity of the obtained weights, it is necessary to introduce CI to test the consistency of the judgment matrix.

(14) $$CI=\frac{\lambda_{\text{max}}-n}{n-1}$$

The greater the value of *CI*, the higher the degree of deviation of the judgment matrix from full consistency; the smaller the value of *CI*, the better the consistency of the matrix; when *CI* takes the value of 0, it indicates that the matrix is fully consistent.

To test whether the judgment matrix has satisfactory consistency, it is necessary to define the test coefficient, *CR*.

(15) $$CR=\frac{CI}{RI}$$

If *CR* < 0.1, the judgment matrix passes the consistency test, and vice versa; the judgment matrix needs to be adjusted until it passes the consistency test, where the Random Index (*RI*) is the Random Consistency Indicator, and *RI* can be obtained from the average Random Consistency Indicator lookup table.

##### 2.1.4.2 Population ageing index

This study constructs an index consisting of the level, scale and structural indicators of population ageing. The development characteristics and differences in population ageing in provincial capital cities and municipalities directly under the central government can be compared. Owing to the differences in the magnitude of the raw data, the indicators were processed with dimensionless data by drawing on existing studies (43). The formula for measuring the composite score was calculated as follows:

(16) $$\left\{ \begin{array}{l} d_{ij}=\frac{x_{ij}-x_{ij\min}}{x_{ij\max}-x_{ij\min}}\left(\mathrm{Positive}\;\mathrm{Indicator}\right)\\ d_{ij}=\frac{x_{ij\max}-x_{ij}}{x_{ij\max}-x_{ij\min}}\left(\mathrm{Negative}\;\mathrm{Indicator}\right) \end{array}\right.$$

(17) $$Z=\overset{m}{\underset{n=1}{\sum }}\left[100\times \left(w_{i}d_{ij}+1/m\right)\right]$$

Here, *Z* is the population ageing index, *dij* represents the evaluation index of population ageing after the dimensionless processing, *xij* represents the original data of population aging index and *xijmin* and *xijmax* represent the minimum and maximum values of the original data, respectively. Where *dij* represents the population ageing index after dimensionless processing, *wi* is the weight determined by the hierarchical analysis and m is the number of evaluation indices. When the standardized value of indicator *xij* takes the minimum value of 0, the comprehensive score of population ageing in a region is 100, and when the standardized value of indicator *xij* takes the maximum value of 1, the comprehensive score of population ageing in a region is 200; that is, the range of the value of the population ageing index *Z* is [100, 200] and the smaller the value of *Z*, the lower the degree of population ageing, and the larger the value of *Z*, the higher the degree of population ageing. The higher values of *Z*.

### 2.2 Data sources

Considering the consistency of data sources, all indicators of population ageing measured by province and city in this article are derived from the 2000 China Population Census Subcounty Data and the 2020 China Population Census Subcounty Data published by China Statistics Press. Owing to the lack of relevant statistics, data for Hong Kong, Macao and Taiwan provinces of China were not included in the statistical analysis.

## 3 Empirical analysis

### 3.1 Analysis of the developmental characteristics of population ageing

#### 3.1.1 Changes in the level of population ageing

Population ageing is the process of increasing the proportion of older persons in the total population and is usually measured by the population ageing rate. The rate of population ageing, regional differences and spatial and temporal patterns are three important indicators that reflect the level of population ageing. The measurement of these three indicators can comprehensively reflect the characteristics of the development of population ageing in the 31 central cities.

The rate of population ageing in cities is generally high and the degree of population ageing continues to increase. The average rate of population ageing in 31 provincial capitals and municipalities directly under the central government rose from 6.91% in 2000 to 11.73% in 2020, an increase of 4.82 percentage points. In 2000, there were 19 cities in the adult stage, 11 in the low-old-age Stage I, 1 in the low-old-age Stage II. By 2020, the number of cities in the adult stage dropped to 1, low-old-age Stage I dropped to 6 and low-old-age Stage II rose to 17, while the number of cities in the middle-old-age Stage I increased to 7 (Table 1). In 2000, the top three cities in terms of population ageing rate were Shanghai (11.46%), Hangzhou (8.83 %) and Shenyang (8.65 %), and the bottom three cities were Lhasa (4.03%), Haikou (4.73%) and Urumqi (4.85%). The top three cities in terms of population ageing rate in 2020 were Chongqing (17.08 %), Shanghai (16.28%) and Shenyang (15.47%), and the bottom three are Lhasa (5.55%), Guangzhou (7.82 %) and Haikou (8.70%).

**Table 1:** Trends in population ageing in 31 central cities, 2000–2020.

Types	Regions							
	2000				2020			
	ER	CR	WR	NER	ER	CR	WR	NER
Young type (par < 4.0%)	0(0.0%)	0(0.0%)	0(0.0%)	0(0.0%)	0(0.0%)	0(0.0%)	0(0.0%)	0(0.0%)
Adult type (4.0–7.0%)	3(15.8%)	4(21.1%)	10(52.6%)	2(10.5%)	0(0.0%)	0(0.0%)	1(100.0%)	0(0.0%)
Low-old-age type I (7.0–10.0%)	6(54.5%)	2(18.2%)	2(18.2%)	1(9.1%)	2(33.3%)	1(16.7%)	3(50.0%)	0(0.0%)
Low-old-age type II (10.0–14.0%)	1(100.0%)	0(0.0%)	0(0.0%)	0(0.0%)	5(29.4%)	5(29.4%)	7(41.2%)	0(0.0%)
Medium-old-age type I (14.0–18.0%)	0(0.0%)	0(0.0%)	0(0.0%)	0(0.0%)	3(42.9%)	0(0.0%)	1(14.3%)	3(42.9%)
Medium-old-age type II (18.0–22.0%)	0(0.0%)	0(0.0%)	0(0.0%)	0(0.0%)	0(0.0%)	0(0.0%)	0(0.0%)	0(0.0%)
Severe-old-age type (par > 22.0%)	0(0.0%)	0(0.0%)	0(0.0%)	0(0.0%)	0(0.0%)	0(0.0%)	0(0.0%)	0(0.0%)

ER: Eastern Region; CR: Central Region; WR: Western Region; NER: North-eastern Region.

From the gap between ageing provinces and cities, urban population ageing experiences higher-than-the-average-level of the province to lower-than-the-average-level of the province’s development process. The average values of population ageing between the 27 provincial capital cities and their host provinces were 6.59% and 6.50%, respectively, in 2000, and became 11.20% and 12.67%, respectively, in 2020; the average population ageing level of the provincial capital city and the province of the gap between the level of population ageing from 0.09 to 1.47%. In 2020, the gap between provincial capital cities and their provinces in terms of population ageing is ranked positively (top 5): Zhengzhou (4.51%), Changsha (3.70%), Chengdu (3.31%), Hefei (3.02%), and Wuhan (2.78%), and negatively (bottom 5): Lhasa (0.12%), Kunming (0.26%), Fuzhou (0.62%), and Guangzhou (0.76%), Yinchuan (0.81%). The gap between the level of population ageing in the cities and that of the provinces in which they are located has widened and is lower than the average level of the province, meaning that the population has continued to concentrate in provincial capitals against the backdrop of low fertility rate, and that the relatively developed public service resources and employment opportunities are an important reason for the concentration of the population in the provincial capitals and the slowing down of population ageing in the provincial capitals. In terms of the spatial pattern of ageing, the degree of ageing of the urban population shows the characteristics of ‘East-Central-North-East–West’ shifting to ‘Northeast-East-Central-West’. In 2000, the population ageing levels of the Northeast, East, Central and Western provinces and municipalities were 6.97, 7.85, 7.00, and 6.08%, respectively. In 2020, the levels of these four regions were 14.76, 12.49, 10.85, and 10.79%, respectively. The Northeast region is one of the areas of increasing population ageing in the country that needs attention. Population ageing is the inevitable result of economic and social development, which is a major concern, owing to the mismatch and imbalance between population ageing and social support system. The Northeast region faces pension problems, amid low fiscal revenues and heavy pension insurance burdens, which, with the rapid aggravation of population ageing, pose serious challenges to the sustainable development of the population. Contrastingly, developed regions on the eastern coast, with a high degree of population ageing, have nothing much to worry about. Despite the seriousness of population ageing in Shanghai, the strong financial support and increasing hardware and software facilities for the older people have pushed it to the forefront in the country in terms of older people’s care services and pension protection, making it a typical model for solving the problem of population ageing. The degree of population ageing in the central and western regions is much lower than that in the north-eastern region, which brings certain opportunities for regional development. However, it is necessary to actively and steadily address issues such as mismatch and imbalance of contradictions between population ageing and social support system.

#### 3.1.2 Structural features of population ageing

Although the indicator of population ageing rate can reflect the overall pattern of the development level of population ageing, it does not reflect the structural characteristics of population ageing. To study population aging, it is necessary not only to observe its development level, but also to analyze its structural characteristics in order to have a more comprehensive view of the problem. The study divides the older adult population into Young- old (65–74 years old), Old- old (75–84 years old) and Oldest- old (85 years old and above), and analyses them from the point of view of the changes in the age structure of the older adult.

In terms of age structure, the size of the older adult population in the 31 central cities during the study period showed a trend of rapid growth, with the proportion of the Young- old population shrinking, the proportion of the Old- old population shrinking slightly, and the proportion of the Oldest- old population expanding, which is intended to show that population ageing is not only manifested in the ageing of the total population, but also in the ageing of the older adult population. From 2000 to 2020, the population sizes of Young- old, Old- old and Oldest- old in the 31 cities increase by 15,438,600, 6,516,300 and 2,837,400 respectively, and the ratio of Young- old, Old- old and Oldest- old in the 31 cities evolves from 69.13:26:42:4.45 in 2000 to 65.00:26.34:8.66 in 2020.The increment of the older adult population size in the municipalities is significant, whether it is Young- old:26:42:4.45. The ratio of Young- old, Old- old and Oldest- old in the 31 cities evolved from 69.13 in 2000 to 65.00:26.34:8.66 in 2020.The increase in the size of the older adult population in the municipalities is significant, and the four municipalities are always in the top 5 in terms of Young- old size, Old- old size and Oldest- old size (Table 2).

**Table 2:** Ranking of the size of the older people population in 2020 (in tens of thousands).

Sorting	Cities	Young-old	Cities	Old-old	Cities	Oldest-old
1	Chongqing	348.71	Chongqing	156.45	Shanghai	45.81
2	Shanghai	266.15	Shanghai	92.94	Chongqing	42.21
3	Beijing	186.32	Chengdu	78.58	Beijing	28.49
4	Chengdu	182.45	Beijing	76.40	Chengdu	24.09
5	Tianjin	138.13	Tianjin	49.96	Tianjin	16.48

The continued deepening of population ageing in municipalities directly under the Central Government is, on the one hand, related to the level of economic development. National central cities such as Beijing and Shanghai, with GDP *per capita* reaching 200,500 yuan (about US$28,500) and 190,700 yuan (about US$27,100) respectively in 2023, are both close to US$30,000, and their strong financial capital is conducive to the construction of the urban habitat and a healthy and long life for the population. In 2022, the life expectancy of the Shanghai population was 83.18 years old, of which 80.84 years old for men and 85.66 years old for women, and the number of older adult people aged 100 years old and above with Shanghai household registration reached 3,528, which is an increase of 19 compared to 2021. On the other hand, the relatively perfect social security and public services, Beijing, Shanghai and other national central cities are not only the country’s economic centre, but also a “welfare highland,” a strong concentration of resources, bringing the wealth effect, public service spill over effect and social security welfare effect, which is conducive to the healthy life of the residents. 2022, Shanghai has a total of 760 older adult care institutions, beds and beds. There are a total of 760 older adult care institutions with 163,600 beds, geriatric medical institutions (geriatric hospitals and geriatric nursing homes) have reached 96, and the number of beds in geriatric nursing homes will be 26,600, an increase of 9.7% compared with that of 2021, the number of people aged 65 years and above under health management will be 2,994,400, accounting for 70.6% of the population of the same age group, and Shanghai has built municipal, district, and street-level Universities for the older adult have been established in Shanghai, and a rich and varied circle of services for older adult life has been constructed, including legal aid for the older adult, associations for the older adult, cultural and artistic troupes for the older adult, and foundations for the older adult, which greatly facilitates the healthy life of the older adult and is conducive to the fulfilment of the value of old age and the extension of healthy life.

In terms of gender structure, the gender ratio of the older adult tends to decrease with age, and the proportion of older women gradually increases, meaning that the phenomenon of female longevity is becoming more pronounced. In 2000, the sex ratio of young-old, old-old and oldest-old in the 31 cities (female = 100, the same below) were 101.82, 82.85, and 59.23, respectively, and in 2020, the sex ratios of the three were 94.06, 83.60 and 73.14, respectively. The biggest change was in the oldest-old sex ratio, and the gap in the oldest-old sex ratio tended to decrease, but the proportion of female oldest-old was still higher. By 2020, the three sex ratios were 94.06, 83.60, and 73.14, respectively, with the largest change in the oldest-old sex ratio. The gap in the oldest-old sex ratio tended to narrow, but the proportion of old-old women remained, and oldest-old women is still larger. From a sub-regional perspective, the young-old sex ratio in 2000 was roughly within the normal range of 105 ± 2, but the young-old sex ratio in 2020 declined significantly, due in part to the first wave of births after the founding of the People’s Republic of China. The oldest-old sex ratio declined markedly in the north-eastern and western cities and rose significantly in the eastern and central cities. The oldest-old sex ratio tended to increase significantly in cities in other regions, except for a significant decline in cities in the Northeast (Table 3).

**Table 3:** Sex ratio of the older population in different age groups, 2000–2020.

Index	Regions					
	Young-old		Old-old		Oldest-old	
	2000	2020	2000	2020	2000	2020
3 North-eastern Cities	100.47	88.20	98.07	78.03	80.16	73.78
10 Eastern Cities	94.81	94.89	72.15	84.11	47.96	66.63
6 Central Cities	102.38	97.53	75.11	87.01	47.96	70.85
12 Western Cities	107.73	93.10	91.84	82.87	69.03	79.55
4 Municipalities	99.26	94.45	84.69	85.09	61.52	74.93
27 Core Cities	102.10	94.02	82.66	83.45	58.99	72.95

In terms of household structure, the proportion of households with older individuals aged 65 and above has been increasing annually, with households with two older people increasing the most. In terms of family structure, the proportion of households with an older adult population aged 65 and above has been rising year by year, with the proportion of households with two older adult persons rising the most. With the gradual decline in the fertility rate, coupled with the further strengthening of the trend of population ageing, the “four-two-one” family structure has gradually become the mainstream, with the “four-two-one” family structure consisting of four grandparents, a married couple and a child, which puts greater pressure on young couples. This family structure puts greater pressure on young couples, who, in addition to supporting the older adult (which is a legacy of Chinese Confucianism and still plays a huge role in today’s Chinese society), also have to raise their children, and so on. Households with older people aged 65 and above accounting for 18.79% in 2000, of which the proportions of households with one older person, two older persons and three or more older people being 13.51, 5.21 and 0.06%, respectively. In 2020, the proportion of households with people aged 65 and above increased to 22.80%, with households with one older person, two and three or more older people being 13.91, 8.76 and 0.13%, respectively. In terms of subregions, the proportion of households with one older person was lowest in north-eastern cities in 2000 but rose the most by 2020, while the proportion of households with one older person in municipalities directly under the Central government remained the highest but rose only marginally by 2020. Municipalities directly under the Central government had the highest proportion of households with one older person and registered the largest increase, while cities in the West had the lowest proportion of households with two older persons. Municipalities continue to have the highest share of households with three or more older persons, whereas Western cities continue to have the lowest share (Table 4).

**Table 4:** Share of older people persons in domestic households in cities in different regions, 2000–2020.

Index	Regions					
	Share of one older people		Share of two older people		Share of three or more older people	
	2000	2020	2000	2020	2000	2020
3 North-eastern Cities	11.96%	15.25%	5.57%	9.69%	0.05%	0.14%
10 Eastern Cities	14.62%	13.73%	6.08%	9.85%	0.08%	0.16%
6 Central Cities	14.34%	14.14%	5.56%	8.78%	0.07%	0.13%
12 Western Cities	12.57%	13.62%	4.23%	7.61%	0.05%	0.10%
4 Municipalities	15.03%	15.48%	6.93%	11.64%	0.08%	0.21%
27 Core Cities	13.29%	13.68%	4.96%	8.33%	0.06%	0.12%

#### 3.1.3 Growth characteristics of population ageing

The tempo of older people age concentration (*TAi*) and tempo of older people geographical concentration (*TGi*) were used to reveal the growth characteristics of all older people: young-old (age 65–74), old-old (75–84) and oldest-old (85 and above).

From the tempo of older people age concentration, there are significant differences in the *TAi* values of different age groups, the mean values of *TAi* for young-old, old-old and oldest-old in 31 central cities are 2.39, 3.00 and 6.45%, respectively, and it is 2.78 for all older people. The maximum *TAi* values for all older people, young-old, old-old and oldest-old in these cities were 4.49% (Harbin), 4.13% (Harbin), 6.27% (Xining) and 8.96% (Xining), respectively. The maximum *TAi* values for all older people, young-old, old-old and oldest-old had the smallest *TAi* values of 1.24% (Guangzhou), 1.00% (Guangzhou), 0.44% (Shanghai) and 3.22% (Lhasa), respectively. In terms of sub-regions, 3 cities in the Northeast region had the highest mean *TAi* for all older people (3.82%), 6 cities in the central region had the lowest mean *TAi* for all older people (2.17%), 12 cities in the Western region had the second highest mean *TAi* for all older people (2.81%) and 10 cities in the Eastern region had the lowest mean *TAi* for all older people had the third highest mean *TAi* value (2.34%). Overall, *TAi* values continued to increase with age. For the young-old, the order was Northeast>West>East>Central; for old-old, Western>Northeast>Central>Eastern and for oldest-old, Northeast>West>Central>East.

From the tempo of older people geographical concentration, there are significant differences in the *TGi* values of different age groups of older people, the mean values of *TGi* for young-old, old-old and oldest-old in the 31 central cities are 0.99, 1.38 and 2.11%, respectively, and the mean value of *TGi* for all older people was 1.10%. The maximum *TGi* values for all older people, young-old, old-old and oldest-old were 5.43% (Haikou), 5.00% (Haikou), 6.24% (Haikou) and 5.86% (Haikou), respectively. The maximum *TGi* values for all older people, young-old, old-old and oldest-old had the smallest *TGi* values of −0.01% (Fuzhou), 0.01% (Taiyuan), −1.23% (Shanghai) and − 0.23% (Lhasa), respectively. In terms of sub-regions, 12 cities in the Western region had the highest mean *TAi* value for all older adults (1.10%), 3 cities in the Northeastern region had the lowest mean *TAi* value (0.88%), 10 cities in the Eastern region registered the second-highest mean *TAi* value (1.01%) and 6 cities in the Central region ranked the second highest mean *TAi* value and had the third highest mean *TAi* value (0.94%). Overall, *TGi* values continued to increase with age. Young-old, Western>Eastern>Northeastern>Central; for the old-old, Western region>Central>Northeast>Eastern; for oldest-old, Western>Central>Eastern>Northeastern.

#### 3.1.4 Characteristics of regional differences in population ageing

The Theil index and *CV* coefficient were used in tandem to reflect the changes in regional differences in population ageing in the 31 central cities (Table 5).

**Table 5:** Measurement of regional differences in population ageing indicators, 2000–2020.

Years	Index					
	Theil index			CV index		
	2000	2020	2020–2000	2000	2020	2020–2000
Old-young ratio	0.0654	0.0655	0.0001	0.3915	0.3762	−0.0154
Population ageing	0.0228	0.0244	0.0016	0.2190	0.2218	0.0028
Size of older people population	0.3597	0.2991	−0.0606	0.9717	0.8666	−0.1051
Young-old population size (age 65–74)	0.3447	0.3011	−0.0436	0.9473	0.8622	−0.0851
Old-old population size (age 75–84)	0.3925	0.2908	−0.1017	1.0228	0.8758	−0.1470
Oldest-old population size (age 85+)	0.4475	0.3339	−0.1136	1.1092	0.9233	−0.1859
Young-old sex ratio	0.0054	0.0011	−0.0043	0.1066	0.0480	−0.0586
Old-old sex ratio	0.0204	0.0029	−0.0175	0.2081	0.0776	−0.1305
Oldest-old sex ratio	0.0433	0.0115	−0.0317	0.3020	0.1542	−0.1478
Share of households with older people	0.0138	0.0166	0.0028	0.1662	0.1808	0.0146
Share of one older people	0.0120	0.0128	0.0008	0.1554	0.1617	0.0063
Share of two older people	0.0317	0.0347	0.0030	0.2530	0.2536	0.0006
Share of three or more older people	0.0997	0.0975	−0.0022	0.4553	0.4561	0.0008

The Theil index and *CV* coefficient of the two indicators, old-age ratio and population ageing level, have major differences and the coefficient of regional difference of the former is much higher than that of the latter, reflecting that the age structure of the population has changed dramatically from 2000 to 2020. The old-age ratio of these cities increased from 0.37 in 2000 to 0.78 in 2020, while population ageing rate rose from 6.91 to 11.73%. The relatively large regional differences in the ratio are mainly due to intra-regional differences in the four major regions, which contributed more than 60% to the Theil index.

According to the indicators, the regional differences in the size of the older population tend to narrow, with the Theil index of the size of the older population reducing from 0.3597 in 2000 to 0.2991 in 2020 and the *CV* coefficient decreasing from 0.9717 to 0.8666, indicating that the size of the older population of 31 cities is steadily increasing and that the regional differences in the size of each other’s older population populations are narrowing. The size of the older population aged 65 and above increased from 16.432 million in 2000 to 41.1987 million in 2020, a 2.51-fold increase. The regional differences in the size of the young-old, old-old and oldest-old populations tend to narrow, and the regional differences in the size of the oldest-old population decreased by the largest margin.

Considering the sex ratio of young-old, old-old and oldest-old, both in 2000 and 2020, the old-age sex ratio increased with age, and its regional differences tended to widen. In 2000, the young-old sex ratio Theil index was 0.0054, old-old sex ratio 0.0204, and oldest-old sex ratio 0.0204. In 2020, the young-old sex ratio Theil index was 0.0011, old-old sex ratio was 0.0029 and oldest-old sex ratio was 0.0115. In 2000–2020, the regional differences in the sex ratios of young-old, old-old and oldest-old individuals tended to decrease, with the largest reduction being in the coefficient of regional differences in the sex ratio of oldest-old.

In terms of the proportion of older people in family households, regional differences in the proportion of households with older people aged 65 and above tended to widen from 2000 to 2020; the Theil index of the cities increased from 0.0138 in 2000 to 0.0166 in 2020; and the *CV* coefficient widened from 0.1662 in 2000 to 0.1808 in 2020. The regional coefficient of variation in the proportion of households with three or more older people was the largest, whereas for households with two or more older people, it was in the middle. The coefficient of regional variation in the share of households with three or more older people is the largest, in households with two older people, it is in the middle, and in households with one older person, it is the smallest, indicating that the regional variation in the share of households with older people aged 65 and above continues to expand as the share of older people in the family household increases.

#### 3.1.5 Quadrant analysis of population ageing indicators

A two-dimensional quadrant analysis was used to classify urban population ageing into different types. This is done by using Population Ageing Rate (PAR) as the horizontal axis, Elderly Population Size (EPS), Proportion of Households with Two or More Elderly Population (PEP), Child Aged Ratio (CAR) and Tempo of Elderly Geographical Concentration (TGi) are the vertical axes, and the origin of the coordinates in the four quadrants are (PAR, EPS), (PAR, PEP), (PAR, CAR), and (PAR, TGi), respectively, which denote the mean value of the two indicators. The first quadrant indicates that a city has a higher than average population ageing on both the horizontal and vertical axes. Quadrant II indicates that a city has a lower than average population ageing on the horizontal axis but higher than average on the vertical axis. Quadrant 3 indicates that a city has an ageing population that is below average on both the horizontal and vertical axes. Quadrant IV indicates that a city has an above-average population ageing on the horizontal axis but a below-average population ageing on the vertical axis.

**Figure 3:**
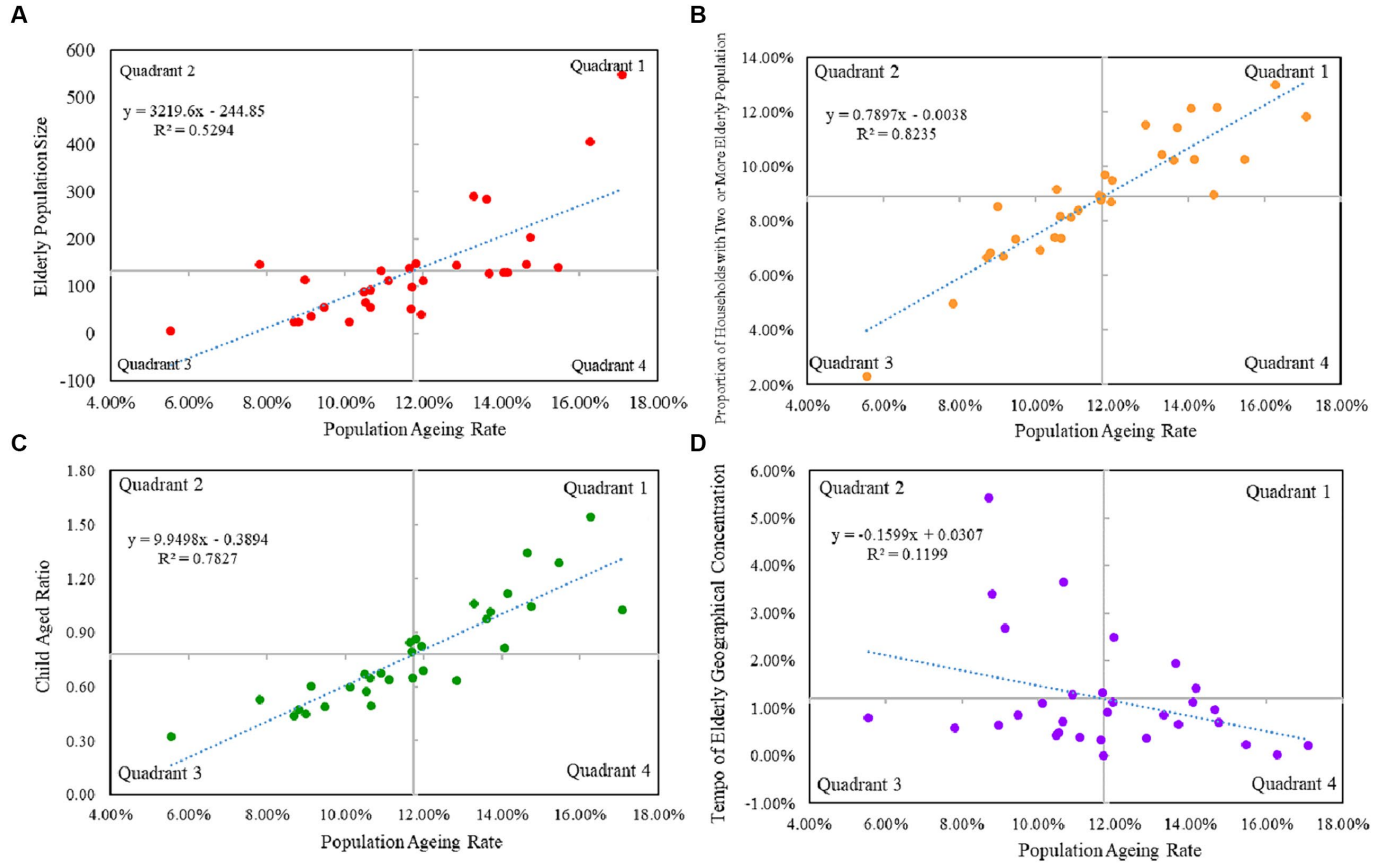
Two-dimensional quadrant analysis of population ageing in 31 cities in China.

From the classification of population ageing level-size structure, the two-dimensional quadrants of Population Ageing Rate (PAR, horizontal axis) and Elderly Population Size (EPS, vertical axis) of 31 cities in 2020 are plotted (Figure 3A), and the results of the measurements are as follows: the first quadrant: Shenyang, Harbin, Beijing, Tianjin, Shijiazhuang, Shanghai, Wuhan, Chongqing, Chengdu and other 9 cities. Quadrant 2: Hangzhou, Guangzhou, Xi’an and 3 other cities. Quadrant 3: 14 cities including Fuzhou, Haikou, Taiyuan, Hefei, Nanchang, Zhengzhou, Nanning, Guiyang, Kunming, Lhasa, Lanzhou, Xining, Yinchuan and Urumqi. Quadrant 4: 5 cities including Changchun, Nanjing, Jinan, Changsha and Hohhot.

From the classification of population ageing level-household structure, the two-dimensional quadrants of Population Ageing Rate (PAR, horizontal axis) and Proportion of Households with Two or More Elderly Population (PEP, vertical axis) for the 31 cities in 2020 are plotted (Figure 3B). The results are as follows: Quadrant 1: 13 cities, including Shenyang, Changchun, Harbin, Beijing, Tianjin, Shijiazhuang, Shanghai, Nanjing, Jinan, Hefei, Wuhan, Chongqing, and Chengdu. Quadrant 2: 3 cities including Hangzhou, Fuzhou and Nanchang. Quadrant 3: 14 cities including Guangzhou, Haikou, Taiyuan, Zhengzhou, Changsha, Nanning, Guiyang, Kunming, Lhasa, Xi’an, Lanzhou, Xining, Yinchuan and Urumqi. Quadrant 4: 1 city, Hohhot.

From the classification of population ageing level-age structure, the two-dimensional quadrant diagrams of Population Ageing Rate (PAR, horizontal axis) and Child Aged Ratio (CAR, vertical axis) of 31 cities in 2020 are plotted (Figure 3C), and the results of the measurements are as follows: Quadrant 1: Shenyang, Changchun, Harbin, Beijing, Tianjin, Shanghai, Nanjing, Jinan, Wuhan Beijing, Tianjin, Shanghai, Nanjing, Jinan, Wuhan, Hohhot, Chongqing, Chengdu and 12 other cities. Quadrant 2: Hangzhou and Lanzhou. Quadrant 3: 15 cities including Shijiazhuang, Fuzhou, Guangzhou, Haikou, Taiyuan, Nanchang, Zhengzhou, Nanning, Guiyang, Kunming, Lhasa, Xi’an, Xining, Yinchuan and Urumqi. Quadrant 4: 2 cities, Hefei and Changsha.

**Figure 4:**
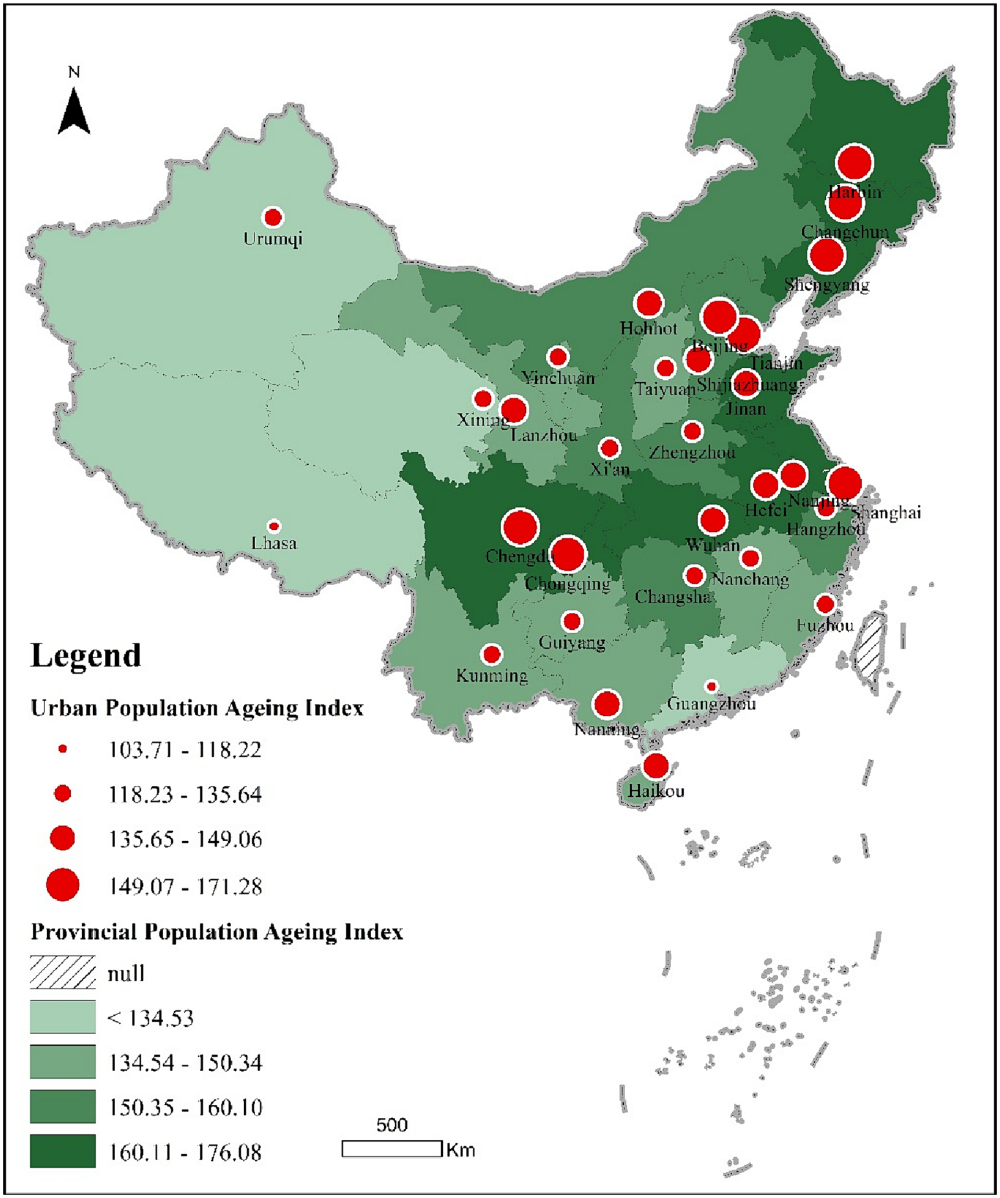
Comprehensive measure of population ageing in 31 Chinese cities and provinces, 2020.

From the classification of Population Ageing Level-Geographic Concentration Rate, the two-dimensional quadrant maps of Population Ageing Rate (PAR, horizontal axis) and Tempo of Elderly Geographical Concentration (TGi, vertical axis) of 31 cities in 2020 are plotted (Figure 3D), and the results of the measurements are as follows: Quadrant I: Changchun, Hefei, and Chengdu and 3 other cities. Quadrant 2: 6 cities including Haikou, Nanning, Xi’an, Lanzhou, Yinchuan, and Urumqi. Quadrant 3: 11 cities including Hangzhou, Fuzhou, Guangzhou, Taiyuan, Nanchang, Zhengzhou, Changsha, Guiyang, Kunming, Lhasa and Nanning. Quadrant 4: 11 cities including Shenyang, Harbin, Beijing, Tianjin, Shijiazhuang, Shanghai, Nanjing, Jinan, Wuhan, Hohhot and Chongqing (Figure 4).

Comparing the four two-dimensional quadrants, it is found that the level of population ageing and the three indicators of size structure, family structure and age structure have to show the characteristics pointed out in the first and the third quadrants. The first quadrant mainly concentrates on the central cities of the coastal provinces and the provinces along the Yangtze River Economic Belt, and the nine central cities distributed in the first quadrant are higher than the average in both the level of population ageing and the size of the older adult population. The third quadrant consists mainly of cities in the central and western regions, and the cities in the third quadrant are lower than average in terms of population aging level, size structure, family structure and age structure. Comparatively speaking, the 2D quadrant classification of PAR-TGi in 2020 mainly shows the characteristics pointed to by the second and fourth quadrants, and the Pearson correlation coefficient of PAR and TGi is 0.35 and shows a negative correlation, which means that with the deepening of the degree of population aging, the TGi of the cities shows a tendency to weaken. The six cities in the second quadrant have population ageing levels below the average. Quadrant 4 mainly focuses on the central cities of coastal province and provinces along the Yangtze River Economic Belt. Eleven cities in the fourth quadrant have higher than average population aging levels, but lower than average Tempo of Elderly Geographical Concentration.

### 3.2 Comprehensive measurement of spatial and temporal patterns of population ageing

#### 3.2.1 Indicator weights

Considering that population ageing is a complex process, describing, analysing and measuring it should be based on a system of indicators, rather than just using a single indicator of ‘the proportion of older people in the total population is increasing’. The population ageing index in this article is a comprehensive indicator to measure the degree of ageing, which is constructed using seven indicators—population ageing level, proportion of older people aged 85 and above, proportion of households with two or more older people, old-age ratio, size of the older people population, *TAi* and *TGi*, aiming to comprehensively reflect the degree of population ageing in the region from the perspective of scale, structure, proportion and other aspects.

First, AHP was used to determine the weights of the evaluation indicators (Table 6).

**Table 6:** Weights of the composite indicator system for population ageing.

Index	Weights
Old-young ratio	0.1352
Level of population ageing	0.1591
Size of the older people population aged 65 and over	0.1561
Proportion of population aged 85 and over	0.1321
Proportion of households with two or more older people	0.1311
Tempo of older people age concentration	0.1442
Tempo of older people geographical concentration	0.1422

Second, to facilitate the comparison between indicators, each indicator was standardized to 100 and above, with a value of 100 demonstrating that all indicators of population ageing are the lowest among the 31 cities, and a value of 200 showing that all indicators of population ageing are the highest among the 31 cities. Similarly, for the 31 provincial units, a value of 100 means that all indicators of population ageing are the lowest among the 31 provincial units, and a value of 200 means that all indicators of population ageing are the highest among the 31 provincial units.

#### 3.2.2 Results and analyses

In terms of city scores, there are two cities with population ageing indexes 103.71–118.22, namely Lhasa and Guangzhou. Twelve cities with population ageing indexes 118.23–135.64 are, from low to high, Zhengzhou, Guiyang, Changsha, Yinchuan, Kunming, Xining, Nanchang, Taiyuan, Urumqi, Fuzhou, Hangzhou and Xi’an. Cities with a population ageing index between 135.65 and 149.06 are, from low to high, Nanning, Hohhot, Haikou, Lanzhou, Wuhan, Shijiazhuang, Hefei, Nanjing and eight other cities. Cities with population ageing indexes between 149.07 and 171.28 are, from low to high, Jinan, Beijing, Chengdu, Shenyang, Tianjin, Changchun, Harbin, Shanghai, Chongqing and another nine cities.

In terms of provincial scores, provinces with a population ageing index lower than 134.53 are, from low to high, Tibet, Xinjiang, Qinghai and Guangdong. For provinces with a population ageing index between 134.54 and 150.34, the scores, from lowest to highest, are from Yunnan, Fujian, Ningxia, Guizhou, Guangxi, Jiangxi, Gansu, Shanxi and eight other provincial units. For provinces with population ageing indexes 150.35–160.10, the scores from low to high are Zhejiang, Shaanxi, Inner Mongolia, Beijing, Henan, Hebei, Tianjin, Hunan and eight other provincial units. For provinces with a population ageing index between 160.11 and 176.08, the scores, from lowest to highest, were in 10 provincial units, including Anhui, Hubei, Jilin, Chongqing, Shanghai, Shandong, Heilongjiang, Jiangsu, Liaoning and Sichuan.

A comparison between the average levels of population ageing in central cities and provinces in which they are located reveals that provincial units generally have higher levels of population ageing than the central cities in the provinces; essentially, the level of population ageing in the central cities is marginally lower than that in the provinces in which they are located. Considering the variability of the data and the relativity of the indicator system, Beijing, Shanghai, Tianjin and Chongqing are treated as being measured by province or city. However, the population ageing indices of 27 central cities differ significantly from those of their host provinces. There are 23 central cities with population ageing indices higher than the average of their provinces, and four central cities with population ageing indices lower than the average of their provinces. The provinces (cities) where the provincial and city population ageing indices are 20.11–35.78 are, in descending order, Henan (Zhengzhou), Hunan (Changsha), Jiangsu (Nanjing), Sichuan (Chengdu), Liaoning (Shenyang), Hubei (Wuhan) and six others. The provinces (cities) with a difference of 15.33–19.05 are, in descending order, Shandong (Jinan), Shaanxi (Xi’an), Shanxi (Taiyuan), Zhejiang (Hangzhou), Anhui (Hefei), Guangdong (Guangzhou), Guizhou (Guiyang) and seven others. The provinces (cities) where the difference between the two is 10.14–14.91 are, in descending order, Hebei (Shijiazhuang), Inner Mongolia (Hohhot), Jiangxi (Nanchang), Heilongjiang (Harbin), Ningxia (Yinchuan) and five others. Provinces (cities) with indices 3.06–9.71 from highest to lowest were Jilin (Changchun), Gansu (Lanzhou), Yunnan (Kunming), Fujian (Fuzhou), Guangxi (Nanning) and another five. The difference between the two is −8.29–2.77 provinces (cities) from high to low is Qinghai (Xining), Tibet (Lhasa), Hainan (Haikou), Xinjiang (Urumqi) and four others.

Overall, the degree of population ageing in China’s 31 central cities shows obvious spatial differentiation. Whether from the two-dimensional quadrant analysis of population ageing indicators or from the composite score of the population ageing index, China’s population ageing has a T-shaped spatial distribution characteristic pointing to the coastal-riverine direction. In the spatial distribution of the Northeast, the Shandong Peninsula, Jiangsu, Shanghai, Zhejiang, Anhui, Hubei, Chongqing, Sichuan and other habitable zones, the degree of population ageing is particularly serious. From the perspective of Chongqing, Shanghai, Harbin, Changchun, Tianjin, Shenyang, Chengdu, Beijing, Jinan and nine others, the degree of population ageing is particularly prominent. In Chongqing, Shanghai and Harbin, population ageing level was 17.08, 16.28 and 14.65%, respectively, in 2020, far higher than the average value of 31 cities (7.82%). Population ageing involves changes in the age structure of a population, which is governed by the laws of demographic transition, with birth and death rates being the two fundamental constraints. In the rapid urbanization stage, population migration has had a significant impact on the ageing of the urban population. For example, Shanghai entered an ageing population as early as 1979, but population migration caused by rapid urbanization has delayed its ageing population. In recent years, with the early arrival of negative population growth in China and the slowing of population migration in the later stages of rapid urbanization, population growth has entered an era of stock. While central cities are attempting to attract people, the trend of population ageing, exacerbated by negative population growth, is irreversible. Our study found that the influences of population ageing are not only affected by three main factors such as birth, death and population migration, but also by location. China’s coastal areas and the Yangtze River Economic Belt are not only economically-developed regions but also have favorable habitats, suitable regional climates and high population densities. The habitat and population systems (births, deaths and migration) interact and constrain each other, and together, they influence regional population ageing.

## 4 Discussion

Geographic systems contain population densities and population age structures of different sizes, and inevitably there are regional differences in population ageing. Wu et al. (21) reveals that China’s population ageing was in the stage of primary ageing and deepening from 2000 to 2010, with regional differences narrowing, with a lower growth rate in the eastern region, and a rapid increase in the burden of ageing and old-age care in central and western regions (35), Ao and Chang (44) found that Baotou, Inner Mongolia to Tengchong, Yunnan is a clearer spatial demarcation line of population ageing in Chinese counties, with high-value counties concentrated in the southeast, low-value counties concentrated in the centre, and generally lower rates of population ageing in the north-west. Our study shares common features with those of the previous two scholars in that there are regional differences in population ageing and the level of population ageing is increasing. The differences are: first, in terms of time span, the two scholars used data from 2000 and 2010, while we used data from 2000 and 2020; second, in terms of measurement indicators, the two scholars used a single indicator of population ageing rate, while we comprehensively analyzed population aging in 31 core cities in China from a number of perspectives, including structure, level, size, speed, and a combination of indicators.

However, there are also shortcomings in our article. The first shortcoming is that the sample is limited, and it is only a spatial and temporal analysis of population ageing in 31 cities in China. There are 333 prefectural-level cities and more than 2,800 county-level cities in China, so at most, it reflects the population ageing situation in the 31 central cities, and the overall overview of China’s population ageing is not sufficiently reflected. The second shortcoming is that the ageing population of the 31 cities may be underestimated. As China’s economic centres, the 31 central cities are also “welfare highlands,” with comprehensive public services and job opportunities, attracting a large number of young people, which, due to the diluting effect of the young people, has led to a possible “underestimation” of the ageing population in the 31 Cities. The third shortcoming is that we have not yet empirically analyzed the impact mechanism of population ageing in the 31 central cities. Although we agree with Ao and Chang (44) that demographic mechanisms are the fundamental driving force behind the evolution of regional differences in population ageing, whether we can reveal the demographic dynamics of the spatial and temporal evolution of population ageing in the 31 central cities is a research question that deserves to be further explored.

## 5 Conclusion and implications

### 5.1 Conclusion

The article comprehensively measures the development characteristics of population ageing in 31 Chinese central cities and classifies them into types by using the population ageing index growth model, the Theil index, the coefficient of variation, the population ageing index and other methods. The study concludes that there is a clear trend of population ageing in China’s 31 central cities, with obvious regional spatial differences, and that China’s population ageing has a T-shaped spatial distribution characteristic pointing along the coast and along the river. Specifically: (1) from 2000 to 2020, the population aging rate of the 31 central cities will generally rise, and the degree of population aging in the cities will show the characteristics of “East-Central-North-East–West” to “North-East–East-Central-West” decreasing. With the increase of age, the TAi value of population aging is increasing, the proportion of young-old in the older adult population is shrinking, the proportion of oldest-old is expanding, and the phenomenon of women’s longevity is more and more significant. (2) Regional differences in the ratio of Young- old to Oldest- old are relatively high, while regional differences in the level of population ageing are relatively small. The level of population aging is classified with the indicators of size structure, family structure and age structure in the first and third quadrants, and with the geographic concentration rate in the second and fourth quadrants. (3) China’s population ageing has a T-shaped spatial distribution characteristic pointing along the coast - along the river, in the spatial distribution of the Northeast, Shandong Peninsula, Jiangsu, Shanghai, Zhejiang, Anhui, Hubei, Chongqing, Sichuan and other liveable zones of the Population ageing is particularly serious.

### 5.2 Implications

Population ageing is a real problem that the world needs to face urgently, and it is a concentrated manifestation of the law of demographic transformation, with far-reaching implications for the sustainable development of human society. The average population ageing rate of China’s 31 provincial capital cities and municipalities directly under the central government will rise from 6.91% in 2000 to 11.73% in 2020, implying that population ageing is moving into the stage of moderate ageing. Geographically, central cities along the “T” corridor between the coast and the Yangtze River have a relatively higher degree of population ageing, meaning that the more economically developed and the more pleasant the environment is, the more prominent the degree of population ageing will be, which will inevitably bring a certain amount of pressure on the city’s economic growth. In response to the increasingly prominent problem of urban population ageing, we offer countermeasures in two areas to cope with the impact of population ageing and to minimize the pressure of population ageing.

Firstly, raising the fertility rate to solve the problem of endogenous growth of urban population ageing. Low fertility is a common problem faced by most countries around the world, including China. In order to address the challenge of low fertility, China needs to take a global view, learn from international experience, accelerate the implementation of the top-level design of the “fertility support policy system,” strengthen the joint efforts of all sectors, eliminate the additional conditions in the current policy system that hinder fertility, adopt policies to encourage fertility, increase the subsidies for fertility, and increase the financial support for families, so as to help those who wish to have children but are afraid to do so. The Government should also provide a series of support measures in terms of finance, time and services for those who wish to have children but are afraid to do so, so as to relieve people’s worries about giving birth to children. It should be noted that China has irreversibly entered the stage of endogenous low fertility, and the policy effect of raising the fertility rate may be small.

Secondly, we should pay attention to the development and utilization of the human resources of the old people, give full play to the value of the old people, and tap the longevity dividend. The development of human resources for the older population is of great significance to the sustainable development of the regional economy, easing the pressure of labor shortages and forming a staggered and complementary employment model with the younger population. The existing problem is that 31 cities have great potential for old people’s labor resources, but their exploitation is limited. Older people are valuable resources for cities, and how to fully tap into them and give full play to their longevity dividend is a common problem faced by the 31 central cities. It is important to note that the development of older people’s human resources is different from that of young people, in that older people’s physical strength and energy will decline as they age, and that it is important to improve older people’s employment practices, labor management and rights and interests in old age, so as to prolong the healthy lifespan of older people, encourage them to adopt healthy lifestyles, prevent disease, prolong their life cycle and alleviate the public health-care pressures of an ageing population.

## Data availability statement

The original contributions presented in the study are included in the article/supplementary material, further inquiries can be directed to the corresponding author.

## Author contributions

LZ: Writing – original draft, Conceptualization. HR: Writing – review & editing, Data curation. CL: Writing – review & editing, Supervision.
